# Long-Term Quality of Life Among Patients Undergoing Endoscopic Pituitary Gland Surgery

**DOI:** 10.3390/jcm13216371

**Published:** 2024-10-24

**Authors:** Narin Nard Carmel Neiderman, Shay Kaufman, Ran Bilaus, Anat Wengier, Tomer Ziv Baran, Avraham Abergel

**Affiliations:** 1 Department of Otolaryngology Head and Neck Surgery and Maxillofacial Surgery, Tel-Aviv Sourasky Medical Center, Affiliated to Tel Aviv University, Tel Aviv 6423906, Israel; carmeln@gmail.com (N.N.C.N.); shaykaufman282@gmail.com (S.K.); ranbar@tlvmc.gov.il (R.B.);; 2 Department of Epidemiology and Preventive Medicine, School of Public Health, Faculty of Medical & Health Sciences, Tel Aviv University, Tel Aviv 6423906, Israel

**Keywords:** quality of life, endoscopic endonasal sinus surgery, pituitary adenoma, skull base, ASBS-Q, SNOT-22

## Abstract

**Introduction/Objectives:** The endoscopic approach to skull base lesions is widely regarded as less invasive and associated with reduced morbidity, offering favorable outcomes, particularly in terms of short-term quality of life (QOL). However, to date, long-term assessments of both nasal function and tumor-related QOL remain limited. To evaluate patients’ long-term nasal- and tumor-related QOL after endoscopic endonasal resection of pituitary tumors and to detect predictors for poor postoperative QOL. Study Design: This study was a prospective cohort study. **Methods:** All patients with pituitary adenomas who underwent trans-sphenoidal surgery at Tel Aviv Sourasky Medical Center between 2014 and 2021 were recruited. Recruited patients completed the Anterior Skull Base Disease-Specific QOL (ASBS-Q) questionnaire and the Sinonasal Outcome Test 22 (SNOT-22) questionnaire before surgery and 1, 2, and 3–5 years after surgery. Clinical data were collected and analyzed. **Results:** The study included 43 patients (18 women). No significant decrease was observed in tumor-related quality of life (QOL) scores, measured by ASBS-Q and SNOT-22, throughout the 5-year follow-up period. SNOT-22 score differences from the preoperative baseline at years 1, 3, and 5 were 0.81 [−4.84–6.58], 3.35 [−4.32–11.02], and 3.73 [−2.22–9.68], respectively, with no statistically significant changes. ASBS-Q scores similarly showed no significant changes over time. Subgroup analyses revealed that tumor characteristics (secreting vs. non-secreting, size), surgical factors (intraoperative cerebrospinal fluid leak, gross tumor resection, use of nasoseptal flap), and endocrine remission did not significantly impact QOL (*p* > 0.05 for all variables). **Conclusions:** Our study demonstrated that patients who underwent endoscopic pituitary lesion resection maintained high nasal- and tumor-related quality of life over a 5-year follow-up period. However, given the limitations of our study, further multi-center studies with larger patient populations are warranted to validate these results.

## 1. Introduction

QOL is a fundamental goal of medical care. Patient-reported outcome measures (PROMs) allow a more objective assessment of the patient’s health-related QOL by providing insight into the patients’ experience with their overall care or healthcare service [1]. PROMs are a cornerstone in the evaluation of surgical interventions, enabling surgeons and healthcare systems to capture meaningful health outcomes from the patient’s perspective. PROMs are essential not only for assessing health-related QoL but also for ensuring high-quality surgical care. Their use is required to fully address patient-centered outcomes in modern surgical practice [2,3].

Benign pituitary gland tumors are associated with pituitary dysfunction and may be accompanied with systemic endocrine changes, higher anxiety-related traits, psychological personality changes [4], and circadian rhythm disorders, resulting in decreased quality of sleep [2,5], therefore deeply impacting mental health and emotional well-being [6]. Benign tumors without endocrine secessional effect may cause headaches or visual disturbances due to pressure on surrounding structures [4]. Seizures, hydrocephalus, and even pituitary apoplexy are less common, but serious presenting signs of a non-secreting adenoma [7].

The endoscopic approach to the anterior skull base caused a revolution in skull base surgery [4], allowing dynamic visualization to be achieved by the endoscope from a wide panoramic to a close-up view and the capability to “look around corners”, expanding the surgical horizons of pituitary surgery [8]. Other major advantage of the endoscopic endonasal approach includes direct anatomical access to a large number of intracranial and paranasal sinus lesions, avoiding the sequelae of a skin incision, facial bone flap or craniotomy, and brain retraction, which is inevitable with conventional neurosurgical incisions, resulting in decreased discomfort, complications, morbidity, mortality, and, indirectly, a decreased length of hospital stay and management costs [6]. Nevertheless, the main flaw is the surgical manipulation of an uninvolved intra-nasal structure to gain adequate access to a functional surgical corridor. As a result, one can expect iatrogenic sinonasal sequelae, which may become part of the disease burden of such patients, and lead to significant physical and psychological post-treatment effects [9,10,11]. It may be expected that the resection of benign skull base tumors via intra-nasal approaches may present an independent cause of significant side effects of surgery in a healthy nose and yield complications that impair the individual’s QOL [12,13,14].

Only a limited number of studies have sought to examine the effect of endoscopic endonasal pituitary adenoma resection on sinonasal- and tumor-related QOL [15,16,17]. The majority of these studies focused on short-term QOL [18].

We had earlier shown that patients reported better overall QOL at 4 to 6 months of follow-up after surgery [18]. The results of two of the Anterior Skull Base Disease-Specific QOL (ASBS-Q) questionnaire subdomains, “pain” and “vitality”, improved significantly and seemed to contribute to overall improved QOL scores [18]. Interestingly, SNOT-22 scores did not deteriorate significantly after surgery, demonstrating that despite extensive nasal surgery, nasal-related QOL was preserved throughout the full duration of the follow-up period [18].

In the current study, we aimed to evaluate whether the trends described in these studies persist after a long-term postoperative follow-up. Our primary aim was to determine the effects of endoscopic/endonasal surgery for pituitary tumors on patient-reported long-term QOLs by means of validated, tumor-specific instruments. Our secondary aim was to identify potential predictors for poor long-term QOL.

## 2. Materials and Methods

### 2.1. Patient Population

All adult patients who underwent endoscopic endonasal surgery for benign pituitary gland tumor resection in our institution in 2014–2021 were enrolled in the study. They all underwent a magnetic resonance imaging (MRI) study prior to surgery. They were also evaluated by a multidisciplinary team, including a neurosurgeon, an endocrinologist, and an otolaryngology expert in rhinology. The same surgeons performed all operations. Patients with prolactinomas underwent surgery as a second line after failure of dopamine agonist therapy or serious adverse effects of medical treatment, while those with other benign tumors underwent surgery as a first-line treatment. An alternative treatment offered by our institution includes radiation therapy to patients with pituitary adenomas who are not candidates for endoscopic surgery or previous surgical failures.

This study was approved by the Institutional Ethics Committee (TLV-0761–19).

### 2.2. Methods

Tumor size was measured on the preoperative MRI scan and classified as microadenoma (<1 cm), macroadenoma (>1 cm) or huge adenoma (>2.5 cm). The histology of each of the lesions was recorded, and the patients were divided into groups of secreting versus non-secreting tumors. We defined “surgical success” as tumors with a mass effect achieving gross tumor resection (GTR), or secretory tumors achieving endocrine remission.

The inclusion criteria were age > 18 years and the ability to understand and consent to responding to the questionnaires. Exclusion criteria were major nasal medical history (e.g., nasal cavity tumor), pregnancy, minors (<16 years), inability to provide informed consent to participate in the study, inability to fill in a questionnaire or inadequacy of responses to the questionnaires (multiple missing answers), and refusal to participate in the study.

Follow-up sessions and self-administered questionnaires were filled in the outpatient clinic at several time points: 1 week pre-operation, 1–5 months, 1–2 years, and 3–5 years. The study population was divided into subgroups according to tumor characteristics, tumor size, intraoperative cerebrospinal fluid (CSF) leak, and reconstruction technique with the aim of identifying patients who were likely to sustain a poor QOL after surgery.

### 2.3. QOL Assessment and Questionnaire

The psychological, social, and physical well-being of the patients were assessed by the ASBS-Q [18], a disease-specific multidimensional questionnaire designed for patients undergoing surgery due to tumors involving the anterior skull base. The questionnaire is self-administered and consists of six domains: the role of performance, physical function, vitality, pain, specific symptoms, and emotional state, yielding a total of 36 questions with a nominal scale of five steps for each question [9]. A higher score means a higher satisfaction. All questions have an identical level of importance.

Patients also filled in a Hebrew-validated Sinonasal Outcome Test 22 (SNOT-22) questionnaire [19]. This questionnaire is a patient-based measurement designed for self-administration, which is commonly administered in the rhinology practice [17,20,21].

### 2.4. Surgical Technique

Extirpation of pituitary gland tumors was performed via an expanded endoscopic approach in all patients. A nasoseptal flap (NSF) was harvested at the beginning of the operation in cases where a high flow CSF leak was anticipated. When dissecting the posterior septal mucosa from the vomer, we usually preserved the posterior septal artery by reflecting down the mucosa of the posterior septum and the choana so that it resembles a minute “rescue flap”. This allows us to elevate an NSF at the end of the tumor resection if an unanticipated high-flow CSF leak occurs. The reconstruction method was tailored to the anticipated defect size and CSF leakage [22]. We preferred to use an NSF prepared in advance with autologous fat when we anticipated a high-flow CSF leak. We used autologous fat for low-flow CSF leaks under clinical consideration

### 2.5. Statistical Analysis

Categorical variables were described as frequency and percentage. Continuous variables were reported as mean and standard deviation. A generalized estimating equation (GEE) model was applied to study the changes over time. The Mann–Whitney test was used to compare the QOL assessment at the specific time point between the two groups. Univariate and multivariable GEE models were used to study the crude and the adjusted values. In addition, an interaction term between time and secretion tumor was added to the model in order to assess whether or not there was a difference in the change over time among patients with secreting and non-secreting tumors. All statistical tests were two-sided, and a *p*-value < 0.05 was accepted as statistically significant. SPSS software (IBM SPSS Statistics for Windows, version 25, IBM corp., Armonk, NY, USA, 2017) was employed for all statistical analyses.

## 3. Results

A total of 43 patients met the inclusion criteria and participated in the study. The questionnaire response rate following surgery was 81.4% at 1–5 months, 67.4% at 1–2 years, and 60.4% at 3–5 years. The mean age of the cohort was 55.93 ± 17.6 years, and 25 (58.1%) were males (Table 1). The patients’ clinical data are shown in Table 1. Preoperatively, 22 (51.2%) patients complained of visual impairments and 26 (60.5%) complained of headaches. Nine (20.9%) patients had microadenomas (<1 cm) and thirty-four (79.1%) had macroadenomas or huge adenomas according to their preoperative MRI findings (Table 1). Fourteen (32.6%) procedures were revision surgeries, and only one patient was operated in our institution prior to initial presentation. Nineteen (44.2%) patients had an intraoperative CSF leak of which eleven (57.9%) were low-flow and eight (42.1%) were high-flow. An NSF was used for reconstruction of a defect in 22 (51.2%) patients. On final pathology, sixteen (37.2%) patients had secreting tumors, twenty-seven (62.8%) had non-secreting tumors, fifteen (34.9%) had adrenocorticotropic hormone-secreting tumors, five (11.6%) had gonadotropic hormone-secreting tumors, four (9.3%) had growth hormone-secreting tumors, and three (7.0%) had lactotroph hormone-secreting tumors (Table 1). Only two (4.7%) patients experienced major postoperative complications: one with meningitis (2.3%) and one with major bleeding (2.3%). Six (14.0%) patients had transient diabetes insipidus (DI) during their postoperative course.

### 3.1. Sinonasal-Related QOL Outcomes

The sinonasal QOL analysis did not demonstrate any significant changes, and there were no deteriorations in SNOT-22 scores at any time points during the postoperative follow-up period (Table 2).

### 3.2. Long-Term Anterior Skull Base Disease-Specific Questionnaire on QOL Outcomes

**Figure 1:**
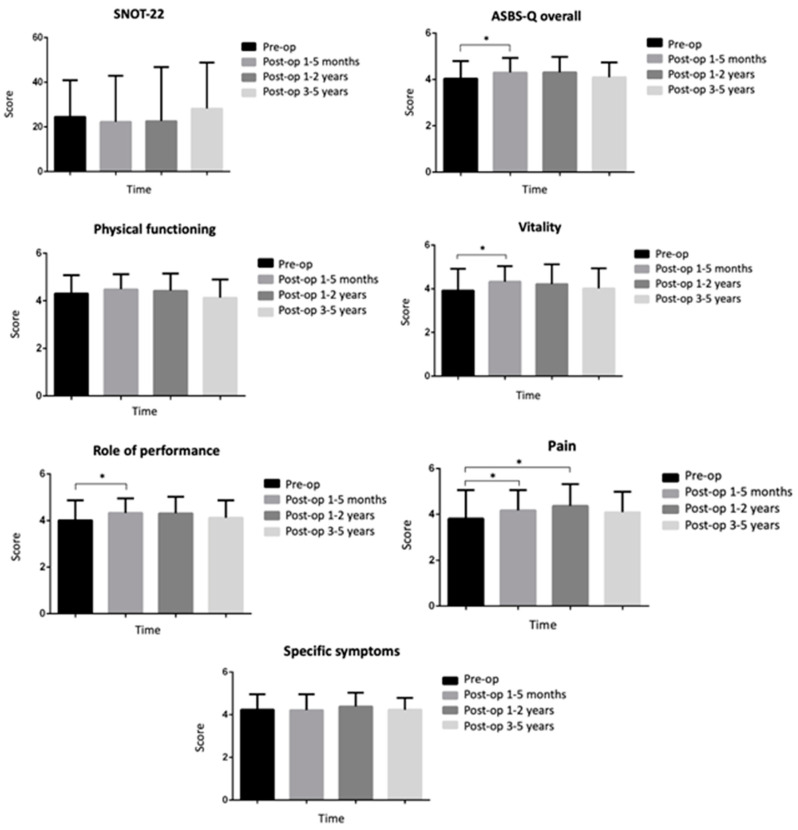
Sinonasal- and tumor-related QOL scores before and after endoscopic pituitary adenoma surgery. Note: the mean and standard deviation questionnaire scores are displayed. Abbreviations: ASBS-Q, Anterior Skull Base Disease-Specific QOL; SNOT-22, Sinonasal Outcome Test 22. * = *p* value < 0.05.

The long-term (1–5 years) overall postoperative QOL analysis did not show any significant deterioration in QOL (*p* = 0.13), while the overall short-term (1–5 months) QOL analysis showed significant improvement, with an increase in the delta score by 0.24 [0.02–0.45], *p* < 0.05 (Table 2). The mean preoperative ASBQ score of 4.04 ± 0.76 was relatively high, while the subdomain scores varied, with the highest being for physical function with a mean of 4.31 ± 0.77 to the lowest being for emotional burden with a mean of 3.67 ± 1.05 (Figure 1). Several subdomain scores contributed to the improvement in the overall QOL score at 1–5 months postoperatively: the “performance-related” subdomain had a subscale delta score of 0.29 [0.04–0.55], *p* < 0.05, the “vitality-related” subdomain had a subscale delta score of 0.37 [0.11–0.64], *p* = 0.05), the “pain-related” subdomain had a subscale delta score of 0.37 [0.02–0.74], *p* < 0.05), and the “emotional state” subdomain had a subscale delta score of 0.49 [0.12–0.79], *p* < 0.05 (Table 2).

The overall QOL did not change significantly at 1–2 years post-surgery (*p* = 0.13), while the only significant improvement among the subdomains was only in the “pain-related” subdomain (subscale delta score of 0.45 [0.09–0.81], *p* < 0.05). The overall QOL also did not change significantly at 3–5 years post-surgery (*p* = 0.37). The “emotional state” subdomain significantly improved by the end of the long-term follow-up (delta score of 0.43 [0.08–0.78], *p* < 0.05).

### 3.3. Subgroup Analysis for Predictors of Postoperative QOL Deterioration

We compared various subgroups within our cohort in order to identify possible predictors of deteriorated QOL following surgery. First, we compared cases of surgical success versus non-success (Table 3). To stress, surgical success was defined as specified in the Methods section (see “Materials and Methods”). We found no significant differences between these two subgroup outcomes except for the “physical function” subdomain at 1–5 months after surgery (short-term), which surprisingly showed better QOL in non-surgical success patients (delta score of 0.64 ± 0.85 vs. −0.09 ± 0.51, *p* < 0.05). No similar significant trend was found in the long-term postoperative follow-up.

We then compared the subgroup of patients who underwent revision surgery to those who did not (Table 4). QOL was better for patients who had revision surgery at 1–2 years after surgery in both the “physical function” subdomain (delta score of 0.51 ± 0.46 vs. −0.16 ± 0.81, *p* < 0.05) and in the “pain” subdomain (delta score of 1.29 ± 1.1 vs. 0.36 ± 0.96, *p* < 0.05).

Comparisons between patients with and without intraoperative CSF leaks (Table 5) failed to detect any deterioration of overall long-term QOL. As expected, QOL was better in patients without a CSF leak. Specifically, the overall ASBS-Q scores were better at 3–5 years after surgery among patients without an intraoperative CSF leak (delta score of 0.21 ± 0.45 versus −0.23 ± 0.58, *p* < 0.05). The “emotional state” subdomain also had better QOL results for the non-CSF leak patients at 3–5 years (delta score of 0.61 ± 0.78 versus −0.18 ± 0.92, *p* < 0.05). As for short-term QOL findings, the “vitality” subdomain was better (delta score of 0.63 ± 0.84 versus 0 ± 0.73, *p* < 0.05) as was the “emotional state” subdomain in the non-CSF leak patients (delta score of 0.76 ± 0.87 versus 0 ± 0.99, *p* < 0.05).

An analysis of the QOL scores among patients who needed intraoperative NSF reconstruction versus those who did not failed to reveal any significant effect of its inclusion on QOL scores over time (Table 6).

We conducted an interaction model to adjust for age, sex, and secretion parameters to detect an over-time effect on QOL (Table 7). The model enabled the assessment of the score difference (delta) at each time point between the groups. There was a significant improvement in overall short-term QOL (delta score of 0.23 [0.02–0.45], *p* < 0.05) at 1–5 months postoperatively, including the “performance-related” subdomain (delta score of 0.29 [0.04–0.54, *p* < 0.05), “vitality” subdomain (delta score of 0.37 [0.11–0.63], *p* < 0.05), the “pain” subdomain (delta score of 0.37 [0.02–0.73], *p* < 0.05), and the “emotional state” subdomain (delta score of 0.49 [0.19–0.79], *p* < 0.05). As for long-term QOL (1–2 years), there was a significant improvement in the “physical function” subdomain interaction with secretion (delta score of 0.53 [0.09–0.96], *p* < 0.05) and in the “pain” subdomain (delta score of 0.45 [0.09–0.81], *p* < 0.05). At 3–5 years postoperatively, there was a significant improvement in the “emotional state” subdomain (delta score of 0.43 [0.09–0.77], *p* < 0.05).

## 4. Discussion

Our study included 43 patients who underwent an extended endoscopic nasal approach for the resection of pituitary adenomas between 2014 and 2021 and maintained a long-term follow-up of up to five years after surgery. The majority of publications evaluating nasal- and tumor-specific quality of life confine their estimation of QOL to the 1st year after surgery. Other longer-term studies primarily focus on general aspects of quality of life (QoL), often using the SF-36 questionnaire as the primary assessment tool [23,24]. Our goal was to evaluate the long-term tumor-related QOL and the nasal-related QOL of patients by identifying and analyzing specific factors likely to affect their QOL.

Despite undergoing major endoscopic nasal surgery, there was no significant decrease in the nasal-related QOL throughout the entire long-term follow-up period (5 years) for the entire study population (n = 43). Pant et al. [15] found that 51 patients who underwent endoscopic pituitary adenoma resection had a temporary decrease in their nasal-related QOL, which improved significantly by 4 to 6 months after surgery and was sustained at 6 to 12 months after surgery. The follow-up period in that study lasted only 24 months. Similarly, McCoul et al. [16] found a significant impairment in a nasal-related QOL 3 weeks after surgery, but a gradual and non-significant improvement from 6 weeks after surgery, lasting until one year after surgery.

We sought to identify which subgroup in our cohort was more likely to sustain nasal-related QOL impairments after surgery. An analysis of our multiple subgroups (Table 3, Table 4, Table 5, Table 6 and Table 7) failed to identify any unique factors decreasing postoperative nasal-related QOL. A regression model also failed to identify any combination of variants affecting overall QOL. Pant et al. [15] and Alobid et al. [17], on the other hand, demonstrated that patients undergoing intraoperative skull base reconstruction with an NSF had poorer nasal-related QOL than those who did not. Different surgical approaches may underlie these findings since pressure on the septal mucosa may result from the manner of harvest from the nasal floor mucosa.

The ASBS-Q questionnaire was used to evaluate the QOL of the entire study population (n = 43), focusing on symptoms unique to skull base tumors and comparing postoperative scores to the scores provided by the patients before surgery. We found that QOL did not deteriorate after a long-term period. According to Pant et al. [15], QOL improved during the postoperative period and throughout approximately one year following surgery. Similarly, McCoul et al. [16] reported a significant improvement in QOL at 6 months and at 1 year after surgery.

Our study is the first to examine long-term QOL after an endoscopic resection of benign skull base tumors. We identified several characteristics that significantly impair QOL over time, including intraoperative CSF leakage. A CSF leak is often associated with tumors that have large diameters and volumes, or those necessitating wide exposure for surgical approaches. Our follow-up at 3–5 years showed that patients with a CSF leak sustained a significant impairment in their overall QOL (*p* = 0.036) and in their emotional state domain (*p* = 0.027). These findings contradict those of McCoul et al. [16], who found that a CSF leak had no impact on QOL after surgery. However, the median follow-up period was only 16 months in their study.

An NSF is often used to reconstruct the base of the skull in cases of a CSF leak. While the NSF reconstruction technique did not impair our patients’ QOL, Pant et al. [15] and Alobid et al. [17] showed that patients who underwent reconstruction by means of an NSF experienced a significant impairment in QOL after the procedure. This raises the possibility that the impairment of QOL was due to the intraoperative CSF leak itself, and not the result of skull reconstruction using an NSF for defect closure.

We analyzed general QOL based upon specific ABSQ’s subdomains. “Pain” was found to improve starting from 1 to 5 months after surgery (*p* = 0.039) and continued to improve for 1–2 years after surgery (*p* = 0.014). We found significant improvement in the “emotional state” subdomain at 1–5 months after surgery (*p* = 0.002), sustaining also at 3–5 years after surgery (*p* = 0.015). We had noted these trends in our earlier study [16], in which we found an improvement in the “emotional state” subdomain at both 2 months and 4–6 months after surgery, attributed to the alleviation of the emotional stress experienced by patients around surgery. We had also found an improvement in the “pain” subdomain at 4–6 months after surgery. The significant long-term improvement in these subdomains is consistent with the findings of McCoul et al. [16], who reported significant improvement in both areas starting from 6 weeks after surgery and lasting for one year afterwards (the end of their follow-up period). Our long-term follow-up shows that the trend of improvement persists beyond one year. Significant improvement in the “emotional state” subdomain can be explained by the relief of anxiety and stress that is known to characterize patients before tumor resection surgery, which also improved after the procedure.

The search for less invasive surgery has not halted and has led to the development of new approaches in endoscopic skull base neurosurgery. The transorbital approach (TOA) represents a valid minimally invasive alternative to a multitude of surgical routes and has the potential to safely address a wide range of pathologies [25]. Combining novel approaches such as TOA may even diminish more the necessity to injure the healthy nose, perhaps yielding even better tumor-specific QOL.

Our study has several possible limitations due to its prospective nature, in a single tertiary institution, with a small cohort size. We had a high rate of revision surgeries, which is attributed to the fact that our center is a large tertiary referral center and considered to deliver high standard of care; therefore, many patients are referrals looking for a second opinion after failures elsewhere, even from other states. We assessed QOL by means of questionnaires, and patients may report an improved QOL to please the attending physician, or alternatively, they may report a poor QOL to gain attention from the attending physician, resulting in reporting bias. Centering on subjective QOL, we did not include an objective assessment of olfaction or postoperative mucociliary clearance in the scope of this study. There may also be a selection bias due to the loss of follow-up in our study. Another potential limitation of our study is the limited number of surgeons performing the endoscopic nasal approach to pituitary adenomas. Moreover, follow-ups were not always accurate due to the clinic’s and patients’ constraints. We did not have access to applicable data regarding QoL outcomes for microscopic or open surgery, making these comparisons beyond the scope of this study. We also neglected other novel and innovative transorbital approaches to the anterior and middle skull base in our study.

## 5. Conclusions

Our study demonstrates that patients who underwent an endoscopic pituitary lesion resection maintained high nasal- and tumor-related quality of life over a 5-year follow-up period. These findings suggest that the endoscopic approach may preserve long-term patient well-being post-surgery. Notably, the patients reported significant improvements in both the “pain” and “emotional state” subdomains of ASBS-Q over the extended follow-up period, indicating potential long-term benefits in these specific areas. However, it is important to acknowledge the limitations of this study, including a relatively small cohort size and a single-center design. To validate these results and further explore potential factors influencing long-term quality of life outcomes in this patient group, multi-center studies with larger patient populations are warranted. Such research could provide more comprehensive insights into the long-term effects of an endoscopic pituitary lesion resection on patient quality of life and help refine surgical approaches and postoperative care strategies.

## Figures and Tables

**Table 1:** Demographic and clinical preoperative, intraoperative, and postoperative characteristics.

Feature		N = 43
Demographic and preoperative background	Female sex	18 (41.9%)
	Age, y	55.93 ± 17.6
	DM type II	6 (14%)
	Hypertension	16 (37.2%)
	**Tumor size**	
	Microadenoma	9 (20.9%)
	Macroadenoma	34 (79.1%)
	Preoperative visual impairments	22 (51.2%)
	Preoperative complaints of headache	26 (60.5%)
	Secreting tumor	16 (37.2%)
	Preoperative endocrine impairment	29 (67.4%)
	**Endocrine impairment (N = 29)**	
	Cushing syndrome	12 (27.9%)
	Acromegaly	4 (9.3%)
	Prolactinoma	5 (11.6%)
	Hypogonadism	4 (9.3%)
	Hypothyroidism	6 (14.0%)
	Addison disease	2 (4.7%)
	Revision surgery	14 (32.6%)
Intraoperative parameters	Intraoperative CSF leak	19 (44.2)
	Intraoperative low-flow CSF leak (N = 19)	11 (57.9%)
	Intraoperative high-flow CSF leak (N = 19)	8 (42.1%)
	NSF reconstruction	22 (51.2%)
Histopathology	Tumor secretion	16 (37.2%)
	Corticotroph adenoma	15 (34.9%)
	Null cell adenoma	16 (37.2%)
	Somatotroph adenoma	4 (9.3%)
	Lactotroph adenoma	3 (7.0%)
	Gonadotroph adenoma	5 (11.6%)
Postoperative complications	Major postoperative complications	2 (4.7)
	Meningitis (N = 2)	1 (2.3%)
	CSF leak (N = 2)	1 (2.3%)
	CSF leak repair (N = 2)	1 (2.3%)
	Intracranial hemorrhage (N = 2)	1 (2.3%)
Postoperative clinical state	Postoperative radiation treatment	6 (14.0%)
	Postoperative transient DI	6 (14.0%)
	Postoperative permanent DI	1 (2.3%)
	GTR in non-secreting tumors (N = 41)	23 (56.1%)
	Endocrine biochemical remission in secreting tumors (N = 33)	22 (66.7%)
	Surgical success	24 (55.8%)

Abbreviations: DM, diabetes mellitus; CSF, cerebrospinal fluids; NSF, nasoseptal flap; DI, diabetes insipidus; GTR, gross tumor resection. Surgical success was defined as achieving GTR or endocrine remission (see Methods).

**Table 2:** Mean difference in questionnaire score before and after surgery (N = 43).

QOL Assessment Instrument	1–5 Months Postoperative (N = 35) Mean Difference [95%CI]	*p*-Value	1–2 Years Postoperative (N = 29) Mean Difference [95%CI]	*p*-Value	3–5 Years Postoperative (N = 26) Mean Difference [95%CI]	*p*-Value
SNOT-22	0.81 [−4.84–6.58]	0.76	3.35 [−4.32–11.02]	0.39	3.73 [−2.22–9.68]	0.22
Overall ASBS-Q	0.24 [0.02–0.45]	**0.029**	0.18 [−0.05–0.404]	0.13	0.101 [−0.12–0.32]	0.37
Role of performance	0.29 [0.04–0.55]	**0.021**	0.23 [−0.04–0.49]	0.101	0.12 [−0.17–0.42]	0.41
Physical function	0.18 [−0.04–0.41]	0.12	0.01 [−0.25–0.28]	0.92	−0.19 [−0.49–0.105]	0.20
Vitality	0.37 [0.11–0.64]	**0.005**	0.27 [−0.03–0.57]	0.082	0.13 [−0.16–0.42]	0.37
Pain	0.37 [0.02–0.74]	**0.039**	0.45 [0.09–0.81]	**0.014**	0.27 [−0.105–0.64]	0.16
Specific symptoms	−0.055 [−0.28–0.17]	0.64	0.09 [−0.115–0.29]	0.39	0.12 [−0.115–0.35]	0.32
Emotional state	0.49 [0.12–0.79]	**0.002**	0.36 [−0.02–0.74]	0.062	0.43 [0.08–0.78]	**0.015**

Abbreviations: ASBS-Q, Anterior Skull Base Disease-Specific QOL; SNOT-22, Sinonasal Outcome Test 22; QOL quality of life; CI = confidence index. Note: **Bold** indicates significant difference in comparison to the preoperative score (*p* < 0.05).

**Table 3:** Comparison between patients who obtained surgical success and those who did not according to SNOT-22 and ASBS-Q questionnaire scores (N = 43).

Variable	Follow-Up Time Point	Success	Failure	*p*-Value
		(N = 24)	(N = 19)	
SNOT-22	1–5 months	2.74 ± 15.58	−4.36 ± 18.41	0.37
	
	1–2 years	0 ± 16.09	8.75 ± 33.91	0.37
	
	3–5 years	6.86 ± 16.92	−0.1 ± 12.9	0.14
	
Overall ASBS-Q	1–5 months	0.06 ± 0.59	0.5 ± 0.76	0.07
	
	1–2 years	0.02 ± 0.62	0.54 ± 0.77	0.13
	
	3–5 years	0.02 ± 0.57	−0.02 ± 0.55	0.69
	
Role of performance	1–5 months	0.09 ± 0.61	0.63 ± 1.04	0.11
	
	1–2 years	0.17 ± 0.76	0.57 ± 0.97	0.39
	
	3–5 years	−0.04 ± 0.77	−0.04 ± 0.74	0.82
	
Physical function	1–5 months	−0.09 ± 0.51	0.64 ± 0.85	**0.007**
	
	1–2 years	−0.22 ± 0.76	0.32 ± 0.75	0.09
	
	3–5 years	−0.38 ± 0.67	0.05 ± 0.87	0.20
	
Vitality	1–5 months	0.23 ± 0.84	0.57 ± 0.84	0.20
	
	1–2 years	0.08 ± 0.92	0.53 ± 1	0.47
	
	3–5 years	0.19 ± 0.77	−0.14 ± 0.77	0.22
	
Pain	1–5 months	0.11 ± 1.27	0.72 ± 0.83	0.08
	
	1–2 years	0.24 ± 0.92	1.08 ± 1.07	0.09
	
	3–5 years	0.31 ± 1.21	−0.1 ± 0.92	0.36
	
Specific symptoms	1–5 months	−0.21 ± 0.66	0.2 ± 0.82	0.18
	
	1–2 years	−0.11 ± 0.55	0.38 ± 0.79	0.13
	
	3–5 years	−0.04 ± 0.7	0.22 ± 0.64	0.36
	
Emotional state	1–5 months	0.4 ± 1.03	0.5 ± 0.93	0.93
	
	1–2 years	0.17 ± 1.08	0.84 ± 1.33	0.15
	
	3–5 years	0.42 ± 0.95	−0.03 ± 0.85	0.15
	

Abbreviations: ASBS-Q, Anterior Skull Base Disease-Specific QOL; SNOT-22, Sinonasal Outcome Test 22. Note: **Bold** indicates significant difference in comparison to the preoperative score (*p* < 0.05).

**Table 4:** Comparison of SNOT-22 and ASBS-Q questionnaire scores between patients who underwent revision surgery and those who underwent primary surgery (N = 43).

Variable	Follow-Up Time Point	Revision Surgery (N = 14) Score Difference from Pre-op	Primary Surgery (N = 29)	*p*-Value
SNOT-22	1–5 months	−1.43 ± 10.75	0.61 ± 18.33	0.96
	
	1–2 years	4.57 ± 23.18	3.26 ± 26.14	0.53
	
	3–5 years	9.4 ± 11.72	0.07 ± 17	0.09
	
Overall ASBS-Q	1–5 months	0.14 ± 0.55	0.25 ± 0.74	0.93
	
	1–2 years	0.63 ± 0.75	0.11 ± 0.68	0.13
	
	3–5 years	−0.08 ± 0.49	0.07 ± 0.6	0.38
	
Role of performance	1–5 months	0.27 ± 0.77	0.3 ± 0.86	0.51
	
	1–2 years	0.9 ± 0.86	0.15 ± 0.8	0.08
	
	3–5 years	−0.08 ± 0.71	−0.01 ± 0.79	0.64
	
Physical function	1–5 months	0 ± 0.8	0.25 ± 0.71	0.46
	
	1–2 years	0.51 ± 0.46	−0.16 ± 0.81	** 0.009 **
	
	3–5 years	−0.19 ± 0.78	−0.23 ± 0.78	0.79
	
Vitality	1–5 months	0.25 ± 0.57	0.4 ± 0.94	0.93
	
	1–2 years	0.57 ± 1.1	0.17 ± 0.93	0.60
	
	3–5 years	−0.03 ± 0.77	0.13 ± 0.79	0.41
	
Pain	1–5 months	0.2 ± 1.02	0.39 ± 1.21	0.39
	
	1–2 years	1.29 ± 1.1	0.36 ± 0.96	** 0.048 **
	
	3–5 years	0.12 ± 1.07	0.18 ± 1.17	0.88
	
Specific symptoms	1–5 months	0.03 ± 0.57	−0.1 ± 0.8	0.55
	
	1–2 years	0.16 ± 0.86	0.07 ± 0.65	0.53
	
	3–5 years	0.03 ± 0.61	0.09 ± 0.75	0.92
	
Emotional state	1–5 months	0.42 ± 0.84	0.44 ± 1.05	0.84
	
	1–2 years	0.84 ± 1.37	0.32 ± 1.16	0.5
	
	3–5 years	−0.02 ± 0.66	0.44 ± 1.05	0.19
	

Abbreviations: ASBS-Q, Anterior Skull Base Disease-Specific QOL; SNOT-22, Sinonasal Outcome Test 22. Note: **Bold** indicates significant difference in comparison to the preoperative score (*p* < 0.05).

**Table 5:** Comparison of SNOT-22 and ASBS-Q questionnaire scores between patients who sustained intraoperative cerebrospinal fluid leak and those who did not (N = 43).

Variable	Follow-Up Time Point	CSF Leak (N = 19)	No CSF Leak (N = 24)	*p*-Value
SNOT-22	1–5 months	3.08 ± 16.99	−1.83 ± 16.73	0.66
	
	1–2 years	5.5 ± 18.21	2.53 ± 28.61	0.95
	
	3–5 years	8.64 ± 16.4	0 ± 14.05	0.23
	
Overall ASBS-Q	1–5 months	−0.04 ± 0.72	0.42 ± 0.6	0.08
	
	1–2 years	0.19 ± 0.87	0.27 ± 0.62	0.61
	
	3–5 years	−0.23 ± 0.58	0.21 ± 0.45	** 0.036 **
	
Role of performance	1–5 months	0.08 ± 1.06	0.45 ± 0.58	0.09
	
	1–2 years	0.31 ± 1.05	0.35 ± 0.74	0.98
	
	3–5 years	−0.3 ± 0.76	0.19 ± 0.67	0.06
	
Physical function	1–5 months	0.03 ± 0.8	0.29 ± 0.68	0.19
	
	1–2 years	−0.01 ± 0.72	0.02 ± 0.86	0.91
	
	3–5 years	−0.34 ± 0.78	−0.11 ± 0.77	0.32
	
Vitality	1–5 months	0 ± 0.73	0.63 ± 0.84	** 0.039 **
	
	1–2 years	0.22 ± 1.22	0.3 ± 0.78	0.95
	
	3–5 years	−0.17 ± 0.75	0.26 ± 0.77	0.19
	
Pain	1–5 months	−0.16 ± 1.04	0.7 ± 1.11	0.11
	
	1–2 years	0.31 ± 1.1	0.78 ± 1	0.39
	
	3–5 years	−0.17 ± 0.93	0.43 ± 1.21	0.25
	
Specific symptoms	1–5 months	−0.15 ± 0.75	0 ± 0.74	0.96
	
	1–2 years	0.08 ± 0.67	0.1 ± 0.73	0.98
	
	3–5 years	−0.02 ± 0.73	0.14 ± 0.64	0.56
	
Emotional state	1–5 months	0 ± 0.99	0.76 ± 0.87	** 0.039 **
	
	1–2 years	0.43 ± 1.33	0.46 ± 1.16	0.98
	
	3–5 years	−0.18 ± 0.92	0.61 ± 0.78	** 0.027 **
	

Abbreviations: ASBS-Q, Anterior Skull Base Disease-Specific QOL; SNOT-22, Sinonasal Outcome Test 22; CSF, cerebrospinal fluid. Note: **Bold** indicates significant difference compared to the preoperative score (*p* < 0.05).

**Table 6:** Comparison of SNOT-22 and ASBS-Q questionnaire scores between patients who underwent intraoperative nasoseptal flap reconstruction and those who did not (N = 43).

Variable	Follow-Up Time Point	NSF Reconstruction (N = 22)	No NSF Reconstruction (N = 21)	*p*-Value
SNOT-22	1–5 months	3.12 ± 16.26	−3.77 ± 17.16	0.36
	
	1–2 years	1.59 ± 18.1	5.38 ± 30	0.82
	
	3–5 years	4.94 ± 16.23	2 ± 14.66	0.57
	
Overall ASBS-Q	1–5 months	0.15 ± 0.72	0.32 ± 0.65	0.96
	
	1–2 years	0.16 ± 0.43	0.29 ± 0.9	0.84
	
	3–5 years	−0.01 ± 0.6	0.03 ± 0.47	0.89
	
Role of performance	1–5 months	0.23 ± 0.94	0.38 ± 0.66	0.73
	
	1–2 years	0.31 ± 0.76	0.36 ± 0.96	0.56
	
	3–5 years	−0.09 ± 0.83	0.07 ± 0.53	0.89
	
Physical function	1–5 months	0.04 ± 0.76	0.37 ± 0.69	0.28
	
	1–2 years	−0.01 ± 0.33	0.02 ± 1.04	0.50
	
	3–5 years	−0.36 ± 0.72	0.11 ± 0.81	0.13
	
Vitality	1–5 months	0.3 ± 0.92	0.43 ± 0.76	0.42
	
	1–2 years	0.24 ± 0.76	0.29 ± 1.13	0.95
	
	3–5 years	0.05 ± 0.84	0.1 ± 0.64	0.93
	
Pain	1–5 months	0.28 ± 1.34	0.4 ± 0.87	0.75
	
	1–2 years	0.49 ± 0.74	0.67 ± 1.27	0.68
	
	3–5 years	0.2 ± 1.12	0.04 ± 1.15	0.98
	
Specific symptoms	1–5 months	−0.1 ± 0.73	−0.01 ± 0.77	0.78
	
	1–2 years	−0.09 ± 0.61	0.24 ± 0.74	0.31
	
	3–5 years	0.12 ± 0.71	−0.06 ± 0.61	0.56
	
Emotional state	1–5 months	0.39 ± 1.07	0.49 ± 0.89	0.93
	
	1–2 years	0.33 ± 1.09	0.55 ± 1.33	0.53
	
	3–5 years	0.3 ± 1.02	0.13 ± 0.7	0.61
	

Abbreviations: ASBS-Q, Anterior Skull Base Disease-Specific QOL; SNOT-22, Sinonasal Outcome Test 22; NSF, Nasoseptal flap.

**Table 7:** Multivariant analysis of the association between overall nasal- and tumor-related quality of life assessment and sex, age, secretion, and time from surgery.

Variable	Parameter	B [CI95%)	*p*-Value
SNOT-22	Female Age Secretion 1–5 m 1–2 y 3–5 y	7.43 [−4.62–19.51] 0.08 [−0.21–0.36] −3.07 [−14.36–8.22] 0.92 [−4.81–6.65] 3.45 [−4.25–11.14] 3.63 [−2.24–9.51]	0.23 0.60 0.59 0.75 0.38 0.23
Overall ASBS-Q	Female Age Secretion 1–5 m 1–2 y 3–5 y	−0.27 [−0.73–0.24] −0.003 [−0.01–0.008] 0.13 [−0.27–0.53] 0.23 [0.02–0.45] 0.17 [−0.05–0.40] 0.11 [−0.12–0.32]	0.32 0.57 0.52 **0.03** 0.13 0.36
Role of performance	Female Age Secretion 1–5 m 1–2 y 3–5 y	−0.24 [−0.73–0.25] −0.002 [−0.01–0.008] 0.21 [−0.20–0.61] 0.29 [0.04–0.54] 0.23 [−0.05–0.49] 0.13 [−0.17–0.42]	0.33 0.66 0.32 **0.02** 0.10 0.39
Physical function	Female Age Secretion 1–5 m 1–2 y 3–5 y	−0.15 [−0.65–0.34] −0.008 [−0.02–0.004] −0.01 [−0.51–0.49] 0.07 [−0.35–0.51] 0.53 [0.09–0.96] 0.21 [−0.36–0.78]	0.55 0.18 0.97 0.73 **0.02** 0.47
Vitality	Female Age Secretion 1–5 m 1–2 y 3–5 y	−0.35 [−0.92–0.22] 0.000 [−0.013–0.012] 0.07 [−0.43–0.56] 0.37 [0.11–0.63] 0.27 [−0.04–0.57] 0.13 [−0.15–0.42]	0.23 0.97 0.79 **0.006** 0.08 0.36
Pain	Female Age Secretion 1–5 m 1–2 y 3–5 y	−0.11 [−0.73–0.51] 0.003 [−0.01–0.02] 0.04 [−0.51–0.59] 0.37 [0.02–0.73] 0.45 [0.09–0.81] 0.27 [−0.13–0.66]	0.73 0.71 0.87 **0.04** **0.01** 0.19
Specific symptoms	Female Age Secretion 1–5 m 1–2 y 3–5 y	−0.22 [−0.62–0.17] −0.006 [−0.02–0.004] 0.04 [−0.31–0.39] −0.05 [−0.28–0.17] 0.09 [−0.12–0.29] 0.12 [−0.11–0.35]	0.27 0.28 0.82 0.63 0.40 0.31
Emotional state	Female Age Secretion 1–5 m 1–2 y 3–5 y	−0.35 [−0.93–0.23] 0.002 [−0.01–0.01] 0.24 [−0.27–0.74] 0.49 [0.19–0.79] 0.36 [−0.01–0.75] 0.43 [0.09–0.77]	0.25 0.80 0.36 **0.002** *0.061* **0.014**

Abbreviations: ASBS-Q, Anterior Skull Base Disease-Specific QOL; SNOT-22, Sinonasal Outcome Test 22. Note: **Bold** indicates significant difference in comparison to the preoperative score (*p* < 0.05), and *italic* indicates borderline significance.

## Data Availability

The data presented in this study are available on request from the corresponding author.
